# Supplementary material for: Novel Receptor Tyrosine Kinase Pathway Inhibitors for Targeted Radionuclide Therapy of Glioblastoma
Source: Pharmaceuticals (Basel). 2021 Jun 29;14(7):626. doi: 10.3390/ph14070626 (PMC8308832; doi:10.3390/ph14070626)
Supplement: Supplementary file 1 [file pharmaceuticals-14-00626-s001.zip › pharmaceuticals-1258516-supplementary.pdf]

Table S1. TKI class radiopharmaceuticals studied in other cancer types

	Radiopharmaceutical	Stage (C/PC)	Reference
**Epidermal growth factor receptor**	[^{89}Zr]Zr- panitumumab	C/PC	[1-5]
	[^{177}Lu]Lu- panitumumab	PC	[6]
	[^{11}C]C-PD153035	C	[7]
	[^{18}F]F-afatinib	C	[8-10]
	[^{89}Zr]Zr- / [^{88}Y]Y / [^{177}Lu]Lu- / [^{111}In]In- / [^{125}I]I- / [^{64}Cu]Cu- cetuximab and [^{111}In]In- / [^{64}Cu]Cu- F(ab′)2 cetuximab	C/PC<br>PC	[6,11-25]
	[^{11}C]C- / [^{18}F]F- / [^{68}Ga]Ga- erlotinib	C	[9,26-37]
	[^{18}F]F- PEGylated anilinoquinazoline derivative (MPG)	C	[38,39]
	[^{11}C]C- / [^{18}F]F- gefinitib	PC	[40,41]
	[^{11}C]C-AZD9291 (osimertinib)	C	[42]
	[^{18}F]F- / [^{124}I]I- / [^{131}I]I- IPQA	PC	[43-47]
	[^{89}Zr]Zr- imgatuzumab (GA201)	PC	[48]
	[^{11}C]C- AZD8931 (EGFR, HER2 and HER3)	synthesis	[49]
	[^{11}C]C- AZD3759	PC	[50]
	[^{124}I]I- SKI 243	PC	[51]
	[^{131}I]I- / [^{99m}Tc]Tc- / [^{68}Ga]Ga- / [^{18}F]F- / [^{111}In]In- EGF	C/PC	[52-55]
	[^{125}I]I- EGFR Fab	PC	[56]
	[^{125}I]I- / [^{111}In]In- ZEGFR:955 affibody	PC	[57,58]
	[^{99m}Tc]Tc- / [^{111}In]In- / [$^{55/57}$Co]Co- / [^{68}Ga]Ga- ZEGFR:2377 affibody	PC	[59-65]
	[^{99m}Tc]Tc- 8B6 nanobody	PC	[66]
	[^{18}F]F- FBEM-Cys-B10 nanofitin	PC	[67]
	[^{89}Zr]Zr- 8709-scFv-Fc vs [^{111}In]In- nimotuzumab	PC	[68]
**Mesenchymal–Epithelial Transition - Hepatocyte growth factor receptor**	[^{18}F]F- AH113804	C	[69]
	[^{89}Zr]Zr- rilotumumab (AMG102)	PC	[70]
	[^{89}Zr]Zr- / [^{76}Br]Br- onartuzumab	PC	[4,71,72]
	[^{89}Zr]Zr- H2 cys-diabody	PC	[73]

	[^{18}F]F- met-pep1	PC	[74,75]
	[^{89}Zr]Zr- / [^{131}I]I- DN30 mAb	PC	[76]
	[^{11}C]C- SU11274	PC	[77]
	[^{125}I]I- anti-c-MET mAb and anti-HGF mAb mixture	PC	[78,79]
	[^{125}I]I- hFab-Met-1	PC	[80]
	[^{125}I]I- mAb (MET4)	PC	[81]
	[^{99m}Tc]Tc- (HYNIC$^{\#}$)- cMBP$^{\%}$	PC	[82,83]
	[^{89}Zr]Zr- PRS-110	PC	[84]
	[^{18}F]F- crizotinib derivative (FPC)	PC	[85]
	[^{99m}Tc]Tc- tricine-EDDA-HYNIC$^{\#}$-c-Met	PC	[86]
**Platelet-derived growth factor receptor**	[^{99m}Tc]Tc- / [^{18}F]F- / [^{124}I]I- imatinib (STI-571) derivatives	PC	[87-89]
	[^{64}Cu]Cu- D13C6 Ab	PC	[90]
	[^{76}Br]Br- / [^{77}Br]Br- PDGFRβ ligands	PC	[91]
	[^{89}Zr]Zr- DFO-ZPDGFRβ affibody conjugate	PC	[92]
	[^{67}Ga]Ga- peptide EG2/4-IPLPPPRRPFFK	PC	[93]
	[^{125}I]I- IQP / IB-IQP $^{€}$	PC	[94]
**Vascular endothelial growth factor receptor**	[^{89}Zr]Zr- / [^{111}In]In- / [^{125}I]I- / [^{131}I]I- bevacizumab<br>[^{89}Zr]Zr- bevacizumab and bevacizumab-IRDye800CW	C/PC	[25,95-102]
	[^{89}Zr]Zr- Df-ranibizumab	PC	[103]
	[^{64}Cu]Cu- NOTA-ramucirumab	PC	[104,105]
	[^{11}C]C- axitinib (AG-013736)	PC	[106]
	[^{11}C]C- PAQ$^{¤}$	PC	[107]
	[^{18}F]F- single-chain VEGF mutants	PC	[108]
	[^{99m}Tc]Tc- HYNIC$^{\#}$-VEGF	PC	[109]
**Fibroblast growth factor receptor**	[^{125}I]I- bFGF mAb	PC	[110]
**Ephrin Receptors**			
EphA3	[^{111}In]In- / [^{213}Bi]Bi- IIIA4	PC	[111,112]
EphB4	[^{64}Cu]Cu- Ab hAb47/hAb131	PC	[113]
	[^{64}Cu]Cu- DOTA-TNYL-RAW peptide	PC	[114]
	[^{111}In]In- TNYL-RAW-CCPM^{+} peptide	PC	[115]

	[18F]F- / [11C]C- indazolylpyrimidine	Syn	[116]
	[18F]F- benzodioxolylpyrimidine	PC	[117]
EphA2	[177Lu]Lu- IF7	PC	[118]
	[64Cu]Cu- 1C1 mAb	PC	[119]
	[89Zr]Zr- DS-8895a	C/PC	[120] (NCT02252211) [121]
	[18F]F- AFP&-SWL peptide	PC	[122]
	[99mTc]Tc- HYNIC#-SWL peptide	PC	[123]
EphB4/EphA2	[18F]F- xanthine	PC	[124]
EphB2	[18F]F- SNEW peptide (SFB-FBAM-AFP-BFP&)	PC	[125]
**Insulin-like growth factor 1 receptor**	[111In]In- / [89Zr]Zr- R1507 mAb and F(ab')2	PC	[126-128]
	[125I]I- des(1-3)IGF-I	PC	[129,130]
	[89Zr]Zr- DFO-1A2G11	PC	[131]
	[111In]In- IGF-1/ IGF-1(E3R)	PC	[132]
	[111In]In- / [99mTc]Tc- ZIGF1R:4551 Affibody	PC	[133-135]
	[18F]F- BMS-754807	PC	[136,137]
	[18F]F- ZSTK474	PC	[138]
**Multi-kinase inhibitors**			
PDGFRα + PDGFRβ + Bcr-Abl + c-FMS + c-Kit	[11C]C- / [124I]I- / [99mTc]Tc- / [18F]F- imatinib	PC	[87-89,139,140]
VEGFR2 + Raf + PDGFR + c-KIT + Flt-3	[11C]C- sorafenib	PC	[141-143]
EGFR + HER2	[18F]F- / [11C]C- lapatinib	C	[144,145]
PDGFR + VEGFR + FLT3 + RET	[18F]F- sunitinib (SU11248)	PC	[146]
PDGFR α/β + FGFR 1-3 + VEGFR 1-3	[11C]C- nintedanib	PC	[106]
c-MET, VEGFR-2, RET, KIT, FLT3, AXL and TEK	[18F]F- cabozantinib (XL-184)	PC	[147]
VEGFR-2 + EGFR + RET	[64Cu]Cu- / [18F]F- vandetanib (ZD6474)	PC	[107,148]
c-MET, EGFR	[89Zr]Zr- DFO- amivantamab	PC	[149]
SRC + KIT + PDGFR + EPHA2 + BCR-ABL fusion	[18F]F- SKI249380 (dasatinib derivative)	C	NCT01916135 [121]
PDGFR + VEGFR + Src + FGFR	[14C]C- ponatinib phase I healthy subjects	C	[150]

(€) IQP and IB-IQP: [125I]-1-{5-iodo-2-[5-(2-methoxyethoxy)-1H-benzo[d]imidazol-1-yl]quinoline-8-yl}piperidin-4-amine ([125I]IIQP) and [125I]-N-3-iodobenzoyl-1-{2-[5-(2-methoxyethoxy)-1H-benzo[d]imidazol-1-yl]quinolin-8-yl}-piperidin-4-amine ([125I]IB-IQP), (%) cMBP (c-Met-binding peptide), (#) HYNIC (hydrazine nicotinamide), (&) N-succinimidyl 4-[18F]fluorobenzoate ([18F]SFB), containing an activated ester for labeling of primary amines, N-(6-(4-[18F]fluorobenzylidene)aminooxyhexyl)-maleimide ([18F]FBAM) for thiol labeling, or 1-(3-azidopropyl)-4-(3-[18F]fluoropropyl)piperazine ([18F]AFP) and 1-(but-3-ynyl)-4-(3-[18F]fluoropropyl) piperazine ([18F]BFP), (+) core-cross-linked polymeric micelles (CCPM), (¤) (R,S)-N-(4-bromo-2-fluorophenyl)-6-methoxy-7-((1-11C-methyl-3-piperidinyl)methoxy)-4-quinazolinamine (PAQ)

References

1. Chang, A.J.; De Silva, R.A.; Lapi, S.E. Development and characterization of 89Zr-labeled panitumumab for immuno-positron emission tomographic imaging of the epidermal growth factor receptor. *Mol Imaging* **2013**, *12*, 17-27.
2. Lindenberg, L.; Adler, S.; Turkbey, I.B.; Mertan, F.; Ton, A.; Do, K.; Kummar, S.; Gonzalez, E.M.; Bhattacharyya, S.; Jacobs, P.M.; et al. Dosimetry and first human experience with (89)Zr-panitumumab. *Am J Nucl Med Mol Imaging* **2017**, *7*, 195-203.
3. Wei, L.; Shi, J.; Afari, G.; Bhattacharyya, S. Preparation of clinical-grade (89) Zr-panitumumab as a positron emission tomography biomarker for evaluating epidermal growth factor receptor-targeted therapy. *J Labelled Comp Radiopharm* **2014**, *57*, 25-35, doi:10.1002/jlcr.3134.
4. Pereira, P.M.R.; Norfleet, J.; Lewis, J.S.; Escorcia, F.E. ImmunoPET Detects Changes in Multi-RTK Tumor Cell Expression Levels in Response to Targeted Kinase Inhibition. *J Nucl Med* **2021**, 62, 366-371. doi:10.2967/jnumed.120.244897.
5. Nayak, T.K.; Garmestani, K.; Milenic, D.E.; Brechbiel, M.W. PET and MRI of metastatic peritoneal and pulmonary colorectal cancer in mice with human epidermal growth factor receptor 1-targeted 89Zr-labeled panitumumab. *J Nucl Med* **2012**, *53*, 113-120, doi:10.2967/jnumed.111.094169.
6. Liu, Z.; Ma, T.; Liu, H.; Jin, Z.; Sun, X.; Zhao, H.; Shi, J.; Jia, B.; Li, F.; Wang, F. 177Lu-labeled antibodies for EGFR-targeted SPECT/CT imaging and radioimmunotherapy in a preclinical head and neck carcinoma model. *Mol Pharm* **2014**, *11*, 800-807, doi:10.1021/mp4005047.
7. Meng, X.; Loo, B.W., Jr.; Ma, L.; Murphy, J.D.; Sun, X.; Yu, J. Molecular imaging with 11C-PD153035 PET/CT predicts survival in non-small cell lung cancer treated with EGFR-TKI: a pilot study. *J Nucl Med* **2011**, *52*, 1573-1579, doi:10.2967/jnumed.111.092874.
8. Slobbe, P.; Windhorst, A.D.; Stigter-van Walsum, M.; Schuit, R.C.; Smit, E.F.; Niessen, H.G.; Solca, F.; Stehle, G.; van Dongen, G.A.; Poot, A.J. Development of [18F]afatinib as new TKI-PET tracer for EGFR positive tumors. *Nucl Med Biol* **2014**, *41*, 749-757, doi:10.1016/j.nucmedbio.2014.06.005.
9. Slobbe, P.; Windhorst, A.D.; Stigter-van Walsum, M.; Smit, E.F.; Niessen, H.G.; Solca, F.; Stehle, G.; van Dongen, G.A.; Poot, A.J. A comparative PET imaging study with the reversible and irreversible EGFR tyrosine kinase inhibitors [(11)C]erlotinib and [(18)F]afatinib in lung cancer-bearing mice. *EJNMMI Res* **2015**, *5*, 14, doi:10.1186/s13550-015-0088-0.
10. van de Stadt, E.A.; Yaqub, M.; Lammertsma, A.A.; Poot, A.J.; Schober, P.R.; Schuit, R.C.; Smit, E.F.; Bahce, I.; Hendrikse, N.H. Quantification of [(18)F]afatinib using PET/CT in NSCLC patients: a feasibility study. *EJNMMI Res* **2020**, *10*, 97, doi:10.1186/s13550-020-00684-4.
11. Menke-van der Houven van Oordt, C.W.; Gootjes, E.C.; Huisman, M.C.; Vugts, D.J.; Roth, C.; Luik, A.M.; Mulder, E.R.; Schuit, R.C.; Boellaard, R.; Hoekstra, O.S.; et al. 89Zr-cetuximab PET imaging in patients with advanced colorectal cancer. *Oncotarget* **2015**, *6*, 30384-30393, doi:10.18632/oncotarget.4672.
12. Makris, N.E.; Boellaard, R.; van Lingen, A.; Lammertsma, A.A.; van Dongen, G.A.; Verheul, H.M.; Menke, C.W.; Huisman, M.C. PET/CT-derived whole-body and bone marrow dosimetry of 89Zr-cetuximab. *J Nucl Med* **2015**, *56*, 249-254, doi:10.2967/jnumed.114.147819.
13. van Helden, E.J.; Elias, S.G.; Gerritse, S.L.; van Es, S.C.; Boon, E.; Huisman, M.C.; van Grieken, N.C.T.; Dekker, H.; van Dongen, G.; Vugts, D.J.; et al. [(89)Zr]Zr-cetuximab PET/CT as biomarker for cetuximab monotherapy in patients with RAS wild-type advanced colorectal cancer. *Eur J Nucl Med Mol Imaging* **2020**, *47*, 849-859, doi:10.1007/s00259-019-04555-6.

14. Even, A.J.; Hamming-Vrieze, O.; van Elmpt, W.; Winnepenninckx, V.J.; Heukelom, J.; Tesselaar, M.E.; Vogel, W.V.; Hoeben, A.; Zegers, C.M.; Vugts, D.J.; et al. Quantitative assessment of Zirconium-89 labeled cetuximab using PET/CT imaging in patients with advanced head and neck cancer: a theragnostic approach. *Oncotarget* **2017**, *8*, 3870-3880, doi:10.18632/oncotarget.13910.
15. McKnight, B.N.; Kim, S.; Boerner, J.L.; Viola, N.T. Cetuximab PET delineated changes in cellular distribution of EGFR upon dasatinib treatment in triple negative breast cancer. *Breast Cancer Res* **2020**, *22*, 37, doi:10.1186/s13058-020-01270-1.
16. Benedetto, R.; Massicano, A.V.F.; Crenshaw, B.K.; Oliveira, R.; Reis, R.M.; Araújo, E.B.; Lapi, S.E. (89)Zr-DFO-Cetuximab as a Molecular Imaging Agent to Identify Cetuximab Resistance in Head and Neck Squamous Cell Carcinoma. *Cancer Biother Radiopharm* **2019**, *34*, 288-296, doi:10.1089/cbr.2018.2616.
17. Perk, L.R.; Visser, G.W.; Vosjan, M.J.; Stigter-van Walsum, M.; Tijink, B.M.; Leemans, C.R.; van Dongen, G.A. (89)Zr as a PET surrogate radioisotope for scouting biodistribution of the therapeutic radiometals (90)Y and (177)Lu in tumor-bearing nude mice after coupling to the internalizing antibody cetuximab. *J Nucl Med* **2005**, *46*, 1898-1906.
18. Bellaye, P.S.; Moreau, M.; Raguin, O.; Oudot, A.; Bernhard, C.; Vrigneaud, J.M.; Dumont, L.; Vandroux, D.; Denat, F.; Cochet, A.; et al. Radiolabeled F(ab')(2)-cetuximab for theranostic purposes in colorectal and skin tumor-bearing mice models. *Clin Transl Oncol* **2018**, *20*, 1557-1570, doi:10.1007/s12094-018-1886-4.
19. Song, I.H.; Lee, T.S.; Park, Y.S.; Lee, J.S.; Lee, B.C.; Moon, B.S.; An, G.I.; Lee, H.W.; Kim, K.I.; Lee, Y.J.; et al. Immuno-PET Imaging and Radioimmunotherapy of 64Cu-/177Lu-Labeled Anti-EGFR Antibody in Esophageal Squamous Cell Carcinoma Model. *J Nucl Med* **2016**, *57*, 1105-1111, doi:10.2967/jnumed.115.167155.
20. Shih, Y.H.; Peng, C.L.; Lee, S.Y.; Chiang, P.F.; Yao, C.J.; Lin, W.J.; Luo, T.Y.; Shieh, M.J. 111In-cetuximab as a diagnostic agent by accessible epidermal growth factor (EGF) receptor targeting in human metastatic colorectal carcinoma. *Oncotarget* **2015**, *6*, 16601-16610, doi:10.18632/oncotarget.3968.
21. van Dijk, L.K.; Hoeben, B.A.; Stegeman, H.; Kaanders, J.H.; Franssen, G.M.; Boerman, O.C.; Bussink, J. 111In-cetuximab-F(ab')2 SPECT imaging for quantification of accessible epidermal growth factor receptors (EGFR) in HNSCC xenografts. *Radiother Oncol* **2013**, *108*, 484-488, doi:10.1016/j.radonc.2013.06.034.
22. van Dijk, L.K.; Yim, C.B.; Franssen, G.M.; Kaanders, J.H.; Rajander, J.; Solin, O.; Grönroos, T.J.; Boerman, O.C.; Bussink, J. PET of EGFR with (64) Cu-cetuximab-F(ab')2 in mice with head and neck squamous cell carcinoma xenografts. *Contrast Media Mol Imaging* **2016**, *11*, 65-70, doi:10.1002/cmmi.1659.
23. Kim, E.J.; Kim, B.S.; Choi, D.B.; Chi, S.G.; Choi, T.H. Enhanced tumor retention of radioiodinated anti-epidermal growth factor receptor antibody using novel bifunctional iodination linker for radioimmunotherapy. *Oncol Rep* **2016**, *35*, 3159-3168, doi:10.3892/or.2016.4706.
24. Niu, G.; Cai, W.; Chen, K.; Chen, X. Non-invasive PET imaging of EGFR degradation induced by a heat shock protein 90 inhibitor. *Mol Imaging Biol* **2008**, *10*, 99-106, doi:10.1007/s11307-007-0123-2.
25. Cohen, R.; Stammes, M.A.; de Roos, I.H.; Stigter-van Walsum, M.; Visser, G.W.; van Dongen, G.A. Inert coupling of IRDye800CW to monoclonal antibodies for clinical optical imaging of tumor targets. *EJNMMI Res* **2011**, *1*, 31, doi:10.1186/2191-219x-1-31.

26. Shamni, O.; Grievink, H.; Itamar, B.; Mishani, E.; Abourbeh, G. Development of a Fluorinated Analogue of Erlotinib for PET Imaging of EGFR Mutation-Positive NSCLC. *Mol Imaging Biol* **2019**, *21*, 696-704, doi:10.1007/s11307-018-1286-8.
27. Memon, A.A.; Jakobsen, S.; Dagnaes-Hansen, F.; Sorensen, B.S.; Keiding, S.; Nexo, E. Positron emission tomography (PET) imaging with [11C]-labeled erlotinib: a micro-PET study on mice with lung tumor xenografts. *Cancer Res* **2009**, *69*, 873-878, doi:10.1158/0008-5472.can-08-3118.
28. Traxl, A.; Beikbaghban, T.; Wanek, T.; Kryeziu, K.; Pirker, C.; Mairinger, S.; Stanek, J.; Filip, T.; Sauberer, M.; Kuntner, C.; et al. [(11)C]Erlotinib PET cannot detect acquired erlotinib resistance in NSCLC tumor xenografts in mice. *Nucl Med Biol* **2017**, *52*, 7-15, doi:10.1016/j.nucmedbio.2017.05.007.
29. Memon, A.A.; Weber, B.; Winterdahl, M.; Jakobsen, S.; Meldgaard, P.; Madsen, H.H.; Keiding, S.; Nexo, E.; Sorensen, B.S. PET imaging of patients with non-small cell lung cancer employing an EGF receptor targeting drug as tracer. *Br J Cancer* **2011**, *105*, 1850-1855.
30. Petrulli, J.R.; Sullivan, J.M.; Zheng, M.Q.; Bennett, D.C.; Charest, J.; Huang, Y.; Morris, E.D.; Contessa, J.N. Quantitative analysis of [11C]-erlotinib PET demonstrates specific binding for activating mutations of the EGFR kinase domain. *Neoplasia* **2013**, *15*, 1347-1353, doi:10.1593/neo.131666.
31. Jain, A.; Kameswaran, M.; Pandey, U.; Prabhash, K.; Sarma, H.D.; Dash, A. (68)Ga labeled Erlotinib: A novel PET probe for imaging EGFR over-expressing tumors. *Bioorg Med Chem Lett* **2017**, *27*, 4552-4557, doi:10.1016/j.bmcl.2017.08.065.
32. Petrulli, J.R.; Hansen, S.B.; Abourbeh, G.; Yaqub, M.; Bahce, I.; Holden, D.; Huang, Y.; Nabulsi, N.B.; Contessa, J.N.; Mishani, E.; et al. A multi species evaluation of the radiation dosimetry of [(11)C]erlotinib, the radiolabeled analog of a clinically utilized tyrosine kinase inhibitor. *Nucl Med Biol* **2017**, *47*, 56-61, doi:10.1016/j.nucmedbio.2016.12.009.
33. Bahce, I.; Yaqub, M.; Errami, H.; Schuit, R.C.; Schober, P.; Thunnissen, E.; Windhorst, A.D.; Lammertsma, A.A.; Smit, E.F.; Hendrikse, N.H. Effects of erlotinib therapy on [(11)C]erlotinib uptake in EGFR mutated, advanced NSCLC. *EJNMMI Res* **2016**, *6*, 10, doi:10.1186/s13550-016-0169-8.
34. Bahce, I.; Smit, E.F.; Lubberink, M.; van der Veldt, A.A.; Yaqub, M.; Windhorst, A.D.; Schuit, R.C.; Thunnissen, E.; Heideman, D.A.; Postmus, P.E.; et al. Development of [(11)C]erlotinib positron emission tomography for in vivo evaluation of EGF receptor mutational status. *Clin Cancer Res* **2013**, *19*, 183-193, doi:10.1158/1078-0432.ccr-12-0289.
35. Yaqub, M.; Bahce, I.; Voorhoeve, C.; Schuit, R.C.; Windhorst, A.D.; Hoekstra, O.S.; Boellaard, R.; Hendrikse, N.H.; Smit, E.F.; Lammertsma, A.A. Quantitative and Simplified Analysis of 11C-Erlotinib Studies. *J Nucl Med* **2016**, *57*, 861-866, doi:10.2967/jnumed.115.165225.
36. Huang, S.; Han, Y.; Chen, M.; Hu, K.; Qi, Y.; Sun, P.; Wang, M.; Wu, H.; Li, G.; Wang, Q.; et al. Radiosynthesis and biological evaluation of (18)F-labeled 4-anilinoquinazoline derivative ((18)F-FEA-Erlotinib) as a potential EGFR PET agent. *Bioorg Med Chem Lett* **2018**, *28*, 1143-1148, doi:10.1016/j.bmcl.2017.08.066.
37. Weber, B.; Winterdahl, M.; Memon, A.; Sorensen, B.S.; Keiding, S.; Sorensen, L.; Nexo, E.; Meldgaard, P. Erlotinib accumulation in brain metastases from non-small cell lung cancer: visualization by positron emission tomography in a patient harboring a mutation in the epidermal growth factor receptor. *J Thorac Oncol* **2011**, *6*, 1287-1289, doi:10.1097/JTO.0b013e318219ab87.
38. Sidaway, P. PET imaging reveals EGFR mutation status. *Nat Rev Clin Oncol* **2018**, *15*, 346, doi:10.1038/s41571-018-0009-z.
39. Sun, X.; Xiao, Z.; Chen, G.; Han, Z.; Liu, Y.; Zhang, C.; Sun, Y.; Song, Y.; Wang, K.; Fang, F.; et al. A PET imaging approach for determining EGFR mutation status for improved lung cancer patient management. *Sci Transl Med* **2018**, *10*, eaan8840, doi:10.1126/scitranslmed.aan8840.

40. Su, H.; Seimbille, Y.; Ferl, G.Z.; Bodenstein, C.; Fueger, B.; Kim, K.J.; Hsu, Y.T.; Dubinett, S.M.; Phelps, M.E.; Czernin, J.; et al. Evaluation of [(18)F]gefitinib as a molecular imaging probe for the assessment of the epidermal growth factor receptor status in malignant tumors. *Eur J Nucl Med Mol Imaging* **2008**, *35*, 1089-1099, doi:10.1007/s00259-007-0636-6.
41. Zhang, M.R.; Kumata, K.; Hatori, A.; Takai, N.; Toyohara, J.; Yamasaki, T.; Yanamoto, K.; Yui, J.; Kawamura, K.; Koike, S.; et al. [11C]Gefitinib ([11c]Iressa): radiosynthesis, in vitro uptake, and in vivo imaging of intact murine fibrosarcoma. *Mol Imaging Biol* **2010**, *12*, 181-191, doi:10.1007/s11307-009-0265-5.
42. Varrone, A.; Varnäs, K.; Jucaite, A.; Cselényi, Z.; Johnström, P.; Schou, M.; Vazquez-Romero, A.; Moein, M.M.; Halldin, C.; Brown, A.P.; et al. A PET study in healthy subjects of brain exposure of (11)C-labelled osimertinib - A drug intended for treatment of brain metastases in non-small cell lung cancer. *J Cereb Blood Flow Metab* **2020**, *40*, 799-807, doi:10.1177/0271678x19843776.
43. Pal, A.; Balatoni, J.A.; Mukhopadhyay, U.; Ogawa, K.; Gonzalez-Lepera, C.; Shavrin, A.; Volgin, A.; Tong, W.; Alauddin, M.M.; Gelovani, J.G. Radiosynthesis and initial in vitro evaluation of [18F]F-PEG6-IPQA-a novel PET radiotracer for imaging EGFR expression-activity in lung carcinomas. *Mol Imaging Biol* **2011**, *13*, 853-861, doi:10.1007/s11307-010-0408-8.
44. Pal, A.; Glekas, A.; Doubrovin, M.; Balatoni, J.; Namavari, M.; Beresten, T.; Maxwell, D.; Soghomonyan, S.; Shavrin, A.; Ageyeva, L.; et al. Molecular imaging of EGFR kinase activity in tumors with 124I-labeled small molecular tracer and positron emission tomography. *Mol Imaging Biol* **2006**, *8*, 262-277, doi:10.1007/s11307-006-0049-0.
45. Yeh, H.H.; Ogawa, K.; Balatoni, J.; Mukhapadhyay, U.; Pal, A.; Gonzalez-Lepera, C.; Shavrin, A.; Soghomonyan, S.; Flores, L., 2nd; Young, D.; et al. Molecular imaging of active mutant L858R EGF receptor (EGFR) kinase-expressing nonsmall cell lung carcinomas using PET/CT. *Proc Natl Acad Sci U S A* **2011**, *108*, 1603-1608, doi:10.1073/pnas.1010744108.
46. Yeh, S.H.; Lin, C.F.; Kong, F.L.; Wang, H.E.; Hsieh, Y.J.; Gelovani, J.G.; Liu, R.S. Molecular imaging of nonsmall cell lung carcinomas expressing active mutant EGFR kinase using PET with [(124)i]-morpholino-IPQA. *Biomed Res Int* **2013**, *2013*, 549359, doi:10.1155/2013/549359.
47. Liu, R.S.; Lin, C.F.; Wang, H.E.; Hsieh, Y.J.; Gelovani, J. Molecular imaging of EGFR kinase activity in lung cancer using radioiodine-labeled IPQA morpholino derivative. *Journal of Nuclear Medicine* **2007**, *48*, 314P.
48. Pool, M.; Kol, A.; Lub-de Hooge, M.N.; Gerdes, C.A.; de Jong, S.; de Vries, E.G.; Terwisscha van Scheltinga, A.G. Extracellular domain shedding influences specific tumor uptake and organ distribution of the EGFR PET tracer 89Zr-imgatuzumab. *Oncotarget* **2016**, *7*, 68111-68121, doi:10.18632/oncotarget.11827.
49. Wang, M.; Gao, M.; Zheng, Q.H. The first radiosynthesis of [(11)C]AZD8931 as a new potential PET agent for imaging of EGFR, HER2 and HER3 signaling. *Bioorg Med Chem Lett* **2014**, *24*, 4455-4459, doi:10.1016/j.bmcl.2014.07.092.
50. Bernard-Gauthier, V.; Bailey, J.J.; Berke, S.; Schirrmacher, R. Recent Advances in the Development and Application of Radiolabeled Kinase Inhibitors for PET Imaging. *Molecules* **2015**, *20*, 22000-22027, doi:10.3390/molecules201219816.
51. Medina, O.P.; Pillarsetty, N.; Glekas, A.; Punzalan, B.; Longo, V.; Gönen, M.; Zanzonico, P.; Smith-Jones, P.; Larson, S.M. Optimizing tumor targeting of the lipophilic EGFR-binding radiotracer SKI 243 using a liposomal nanoparticle delivery system. *J Control Release* **2011**, *149*, 292-298, doi:10.1016/j.jconrel.2010.10.024.

52. Cuartero-Plaza, A.; Martínez-Miralles, E.; Rosell, R.; Vadell-Nadal, C.; Farré, M.; Real, F.X. Radiolocalization of squamous lung carcinoma with 131I-labeled epidermal growth factor. *Clin Cancer Res* **1996**, *2*, 13-20.
53. Rusckowski, M.; Qu, T.; Chang, F.; Hnatowich, D.J. Technetium-99m labeled epidermal growth factor-tumor imaging in mice. *J Pept Res* **1997**, *50*, 393-401, doi:10.1111/j.1399-3011.1997.tb01200.x.
54. Velikyan, I.; Sundberg, A.L.; Lindhe, O.; Höglund, A.U.; Eriksson, O.; Werner, E.; Carlsson, J.; Bergström, M.; Långström, B.; Tolmachev, V. Preparation and evaluation of (68)Ga-DOTA-hEGF for visualization of EGFR expression in malignant tumors. *J Nucl Med* **2005**, *46*, 1881-1888.
55. Reilly, R.M.; Kiarash, R.; Cameron, R.G.; Porlier, N.; Sandhu, J.; Hill, R.P.; Vallis, K.; Hendler, A.; Gariépy, J. 111In-labeled EGF is selectively radiotoxic to human breast cancer cells overexpressing EGFR. *J Nucl Med* **2000**, *41*, 429-438.
56. Xu, N.; Cai, G.; Ye, W.; Wang, X.; Li, Y.; Zhao, P.; Zhang, A.; Zhang, R.; Cao, B. Molecular imaging application of radioiodinated anti-EGFR human Fab to EGFR-overexpressing tumor xenografts. *Anticancer Res* **2009**, *29*, 4005-4011.
57. Nordberg, E.; Orlova, A.; Friedman, M.; Tolmachev, V.; Ståhl, S.; Nilsson, F.Y.; Glimelius, B.; Carlsson, J. In vivo and in vitro uptake of 111In, delivered with the affibody molecule (ZEGFR:955)2, in EGFR expressing tumour cells. *Oncol Rep* **2008**, *19*, 853-857, doi:10.3892/or.19.4.853.
58. Nordberg, E.; Friedman, M.; Göstring, L.; Adams, G.P.; Brismar, H.; Nilsson, F.Y.; Ståhl, S.; Glimelius, B.; Carlsson, J. Cellular studies of binding, internalization and retention of a radiolabeled EGFR-binding affibody molecule. *Nucl Med Biol* **2007**, *34*, 609-618, doi:10.1016/j.nucmedbio.2007.05.010.
59. Andersson, K.G.; Oroujeni, M.; Garousi, J.; Mitran, B.; Ståhl, S.; Orlova, A.; Löfblom, J.; Tolmachev, V. Feasibility of imaging of epidermal growth factor receptor expression with ZEGFR:2377 affibody molecule labeled with 99mTc using a peptide-based cysteine-containing chelator. *Int J Oncol* **2016**, *49*, 2285-2293, doi:10.3892/ijo.2016.3721.
60. Tolmachev, V.; Rosik, D.; Wållberg, H.; Sjöberg, A.; Sandström, M.; Hansson, M.; Wennborg, A.; Orlova, A. Imaging of EGFR expression in murine xenografts using site-specifically labelled anti-EGFR 111In-DOTA-Z EGFR:2377 Affibody molecule: aspect of the injected tracer amount. *Eur J Nucl Med Mol Imaging* **2010**, *37*, 613-622, doi:10.1007/s00259-009-1283-x.
61. Cheng, Q.; Wållberg, H.; Grafström, J.; Lu, L.; Thorell, J.O.; Hägg Olofsson, M.; Linder, S.; Johansson, K.; Tegnebratt, T.; Arnér, E.S.; et al. Preclinical PET imaging of EGFR levels: pairing a targeting with a non-targeting Sel-tagged Affibody-based tracer to estimate the specific uptake. *EJNMMI Res* **2016**, *6*, 58, doi:10.1186/s13550-016-0213-8.
62. Garousi, J.; Andersson, K.G.; Mitran, B.; Pichl, M.L.; Ståhl, S.; Orlova, A.; Löfblom, J.; Tolmachev, V. PET imaging of epidermal growth factor receptor expression in tumours using 89Zr-labelled ZEGFR:2377 affibody molecules. *Int J Oncol* **2016**, *48*, 1325-1332, doi:10.3892/ijo.2016.3369.
63. Oroujeni, M.; Garousi, J.; Andersson, K.G.; Löfblom, J.; Mitran, B.; Orlova, A.; Tolmachev, V. Preclinical Evaluation of [(68)Ga]Ga-DFO-ZEGFR:2377: A Promising Affibody-Based Probe for Noninvasive PET Imaging of EGFR Expression in Tumors. *Cells* **2018**, *7*, doi:10.3390/cells7090141.
64. Mitran, B.; Andersson, K.G.; Lindström, E.; Garousi, J.; Rosestedt, M.; Tolmachev, V.; Ståhl, S.; Orlova, A.; Löfblom, J. Affibody-mediated imaging of EGFR expression in prostate cancer using radiocobalt-labeled DOTA-ZEGFR:2377. *Oncol Rep* **2019**, *41*, 534-542, doi:10.3892/or.2018.6792.
65. Oroujeni, M.; Xu, T.; Gagnon, K.; Rinne, S.S.; Weis, J.; Garousi, J.; Andersson, K.G.; Löfblom, J.; Orlova, A.; Tolmachev, V. The Use of a Non-Conventional Long-Lived Gallium Radioisotope (66)Ga Improves Imaging Contrast of EGFR Expression in Malignant Tumours Using DFO-ZEGFR:2377 Affibody Molecule. *Pharmaceutics* **2021**, *13*, 292, doi:10.3390/pharmaceutics13020292.

66. Huang, L.; Gainkam, L.O.; Caveliers, V.; Vanhove, C.; Keyaerts, M.; De Baetselier, P.; Bossuyt, A.; Revets, H.; Lahoutte, T. SPECT imaging with 99mTc-labeled EGFR-specific nanobody for in vivo monitoring of EGFR expression. *Mol Imaging Biol* **2008**, *10*, 167-175, doi:10.1007/s11307-008-0133-8.
67. Goux, M.; Becker, G.; Gorré, H.; Dammicco, S.; Desselle, A.; Egrise, D.; Leroi, N.; Lallemand, F.; Bahri, M.A.; Doumont, G.; et al. Nanofitin as a New Molecular-Imaging Agent for the Diagnosis of Epidermal Growth Factor Receptor Over-Expressing Tumors. *Bioconjug Chem* **2017**, *28*, 2361-2371, doi:10.1021/acs.bioconjchem.7b00374.
68. Alizadeh, E.; Behlol Ayaz Ahmed, K.; Raja Solomon, V.; Gaja, V.; Bernhard, W.; Makhlouf, A.; Gonzalez, C.; Barreto, K.; Casaco, A.; Geyer, C.R.; et al. (89)Zr-Labeled Domain II-Specific scFv-Fc ImmunoPET Probe for Imaging Epidermal Growth Factor Receptor In Vivo. *Cancers* **2021**, *13*, doi:10.3390/cancers13030560.
69. Arulappu, A.; Battle, M.; Eisenblaetter, M.; McRobbie, G.; Khan, I.; Monypenny, J.; Weitsman, G.; Galazi, M.; Hoppmann, S.; Gazinska, P.; et al. c-Met PET Imaging Detects Early-Stage Locoregional Recurrence of Basal-Like Breast Cancer. *J Nucl Med* **2016**, *57*, 765-770, doi:10.2967/jnumed.115.164384.
70. Price, E.W.; Carnazza, K.E.; Carlin, S.D.; Cho, A.; Edwards, K.J.; Sevak, K.K.; Glaser, J.M.; de Stanchina, E.; Janjigian, Y.Y.; Lewis, J.S. (89)Zr-DFO-AMG102 Immuno-PET to Determine Local Hepatocyte Growth Factor Protein Levels in Tumors for Enhanced Patient Selection. *J Nucl Med* **2017**, *58*, 1386-1394, doi:10.2967/jnumed.116.187310.
71. Jagoda, E.M.; Lang, L.; Bhadrasetty, V.; Histed, S.; Williams, M.; Kramer-Marek, G.; Mena, E.; Rosenblum, L.; Marik, J.; Tinianow, J.N.; et al. Immuno-PET of the hepatocyte growth factor receptor Met using the 1-armed antibody onartuzumab. *J Nucl Med* **2012**, *53*, 1592-1600, doi:10.2967/jnumed.111.102293.
72. Pool, M.; Terwisscha van Scheltinga, A.G.T.; Kol, A.; Giesen, D.; de Vries, E.G.E.; Lub-de Hooge, M.N. (89)Zr-Onartuzumab PET imaging of c-MET receptor dynamics. *Eur J Nucl Med Mol Imaging* **2017**, *44*, 1328-1336, doi:10.1007/s00259-017-3672-x.
73. Li, K.; Tavaré, R.; Zettlitz, K.A.; Mumenthaler, S.M.; Mallick, P.; Zhou, Y.; Marks, J.D.; Wu, A.M. Anti-MET immunoPET for non-small cell lung cancer using novel fully human antibody fragments. *Mol Cancer Ther* **2014**, *13*, 2607-2617, doi:10.1158/1535-7163.mct-14-0363.
74. Zhao, P.; Grabinski, T.; Gao, C.; Skinner, R.S.; Giambernardi, T.; Su, Y.; Hudson, E.; Resau, J.; Gross, M.; Vande Woude, G.F.; et al. Identification of a met-binding peptide from a phage display library. *Clin Cancer Res* **2007**, *13*, 6049-6055, doi:10.1158/1078-0432.ccr-07-0035.
75. Li, W.; Zheng, H.; Xu, J.; Cao, S.; Xu, X.; Xiao, P. Imaging c-Met expression using 18F-labeled binding peptide in human cancer xenografts. *PLoS One* **2018**, *13*, e0199024, doi:10.1371/journal.pone.0199024.
76. Perk, L.R.; Stigter-van Walsum, M.; Visser, G.W.; Kloet, R.W.; Vosjan, M.J.; Leemans, C.R.; Giaccone, G.; Albano, R.; Comoglio, P.M.; van Dongen, G.A. Quantitative PET imaging of Met-expressing human cancer xenografts with 89Zr-labelled monoclonal antibody DN30. *Eur J Nucl Med Mol Imaging* **2008**, *35*, 1857-1867, doi:10.1007/s00259-008-0774-5.
77. Wu, C.; Tang, Z.; Fan, W.; Zhu, W.; Wang, C.; Somoza, E.; Owino, N.; Li, R.; Ma, P.C.; Wang, Y. In vivo positron emission tomography (PET) imaging of mesenchymal-epithelial transition (MET) receptor. *J Med Chem* **2010**, *53*, 139-146, doi:10.1021/jm900803q.
78. Hay, R.V.; Cao, B.; Skinner, R.S.; Su, Y.; Zhao, P.; Gustafson, M.F.; Qian, C.N.; Teh, B.T.; Knudsen, B.S.; Resau, J.H.; et al. Nuclear imaging of Met-expressing human and canine cancer xenografts with radiolabeled monoclonal antibodies (MetSeek). *Clin Cancer Res* **2005**, *11*, 7064s-7069s, doi:10.1158/1078-0432.ccr-1004-0014.

79. Hay, R.V.; Cao, B.; Skinner, R.S.; Wang, L.M.; Su, Y.; Resau, J.H.; Vande Woude, G.F.; Gross, M.D. Radioimmunoscintigraphy of tumors autocrine for human met and hepatocyte growth factor/scatter factor. *Mol Imaging* **2002**, *1*, 56-62, doi:10.1162/153535002753395716.
80. Jiao, Y.; Zhao, P.; Zhu, J.; Grabinski, T.; Feng, Z.; Guan, X.; Skinner, R.S.; Gross, M.D.; Hay, R.V.; Tachibana, H.; et al. Construction of human naïve Fab library and characterization of anti-met Fab fragment generated from the library. *Mol Biotechnol* **2005**, *31*, 41-54, doi:10.1385/mb:31:1:041.
81. Knudsen, B.S.; Zhao, P.; Resau, J.; Cottingham, S.; Gherardi, E.; Xu, E.; Berghuis, B.; Daugherty, J.; Grabinski, T.; Toro, J.; et al. A novel multipurpose monoclonal antibody for evaluating human c-Met expression in preclinical and clinical settings. *Appl Immunohistochem Mol Morphol* **2009**, *17*, 57-67, doi:10.1097/PAI.0b013e3181816ae2.
82. Jagoda, E.M.; Bhattacharyya, S.; Kalen, J.; Riffle, L.; Leeder, A.; Histed, S.; Williams, M.; Wong, K.J.; Xu, B.; Szajek, L.P.; et al. Imaging the Met Receptor Tyrosine Kinase (Met) and Assessing Tumor Responses to a Met Tyrosine Kinase Inhibitor in Human Xenograft Mouse Models with a [99mTc] (AH-113018) or Cy 5** (AH-112543) Labeled Peptide. *Mol Imaging* **2015**, *14*, 499-515.
83. Han, Z.; Xiao, Y.; Wang, K.; Yan, J.; Xiao, Z.; Fang, F.; Jin, Z.; Liu, Y.; Sun, X.; Shen, B. Development of a SPECT Tracer to Image c-Met Expression in a Xenograft Model of Non-Small Cell Lung Cancer. *J Nucl Med* **2018**, *59*, 1686-1691, doi:10.2967/jnumed.117.206730.
84. Terwisscha van Scheltinga, A.G.; Lub-de Hooge, M.N.; Hinner, M.J.; Verheijen, R.B.; Allersdorfer, A.; Hülsmeyer, M.; Nagengast, W.B.; Schröder, C.P.; Kosterink, J.G.; de Vries, E.G.; et al. In vivo visualization of MET tumor expression and anticalin biodistribution with the MET-specific anticalin 89Zr-PRS-110 PET tracer. *J Nucl Med* **2014**, *55*, 665-671, doi:10.2967/jnumed.113.124941.
85. Lin, Q.; Zhang, Y.; Fu, Z.; Hu, B.; Si, Z.; Zhao, Y.; Shi, H.; Cheng, D. Synthesis and evaluation of (18)F labeled crizotinib derivative [(18)F]FPC as a novel PET probe for imaging c-MET-positive NSCLC tumor. *Bioorg Med Chem* **2020**, *28*, 115577, doi:10.1016/j.bmc.2020.115577.
86. Zhang, Y.; Meng, L.; Li, B.; Tan, H.; Lin, Q.Y.; Cheng, D.; Shi, H. Preliminary application of micro-SPECT/CT imaging by (99m) Tc-tricine-EDDA-HYNIC-c-Met for non-small-cell lung cancer. *Chem Biol Drug Des* **2019**, *93*, 447-453, doi:10.1111/cbdd.13432.
87. Gundogdu, E.; Demir, E.S.; Özgenç, E.; Yeğen, G.; Aksu, B. Applying Quality by Design Principles in the Development and Preparation of a New Radiopharmaceutical: Technetium-99m-Imatinib Mesylate. *ACS Omega* **2020**, *5*, 5297-5305, doi:10.1021/acsomega.9b04327.
88. Doubrovin, M.; Kochetkova, T.; Santos, E.; Veach, D.R.; Smith-Jones, P.; Pillarsetty, N.; Balatoni, J.; Bornmann, W.; Gelovani, J.; Larson, S.M. (124)I-iodopyridopyrimidinone for PET of Abl kinase-expressing tumors in vivo. *J Nucl Med* **2010**, *51*, 121-129, doi:10.2967/jnumed.109.066126.
89. Glekas, A.P.; Pillarsetty, N.K.; Punzalan, B.; Khan, N.; Smith-Jones, P.; Larson, S.M. In vivo imaging of Bcr-Abl overexpressing tumors with a radiolabeled imatinib analog as an imaging surrogate for imatinib. *J Nucl Med* **2011**, *52*, 1301-1307, doi:10.2967/jnumed.110.085050.
90. Wagner, M.; Wuest, M.; Hamann, I.; Lopez-Campistrous, A.; McMullen, T.P.W.; Wuest, F. Molecular imaging of platelet-derived growth factor receptor-alpha (PDGFRα) in papillary thyroid cancer using immuno-PET. *Nucl Med Biol* **2018**, *58*, 51-58, doi:10.1016/j.nucmedbio.2017.12.005.
91. Effendi, N.; Mishiro, K.; Takarada, T.; Makino, A.; Yamada, D.; Kitamura, Y.; Shiba, K.; Kiyono, Y.; Odani, A.; Ogawa, K. Radiobrominated benzimidazole-quinoline derivatives as Platelet-derived growth factor receptor beta (PDGFRβ) imaging probes. *Sci Rep* **2018**, *8*, 10369, doi:10.1038/s41598-018-28529-0.
92. Cai, H.; Shi, Q.; Tang, Y.; Chen, L.; Chen, Y.; Tao, Z.; Yang, H.; Xie, F.; Wu, X.; Liu, N.; et al. Positron Emission Tomography Imaging of Platelet-Derived Growth Factor Receptor β in Colorectal Tumor Xenograft Using Zirconium-89 Labeled Dimeric Affibody Molecule. *Mol Pharm* **2019**, *16*, 1950-1957, doi:10.1021/acs.molpharmaceut.8b01317.

93. Effendi, N.; Mishiro, K.; Shiba, K.; Kinuya, S.; Ogawa, K. Development of Radiogallium-Labeled Peptides for Platelet-Derived Growth Factor Receptor β (PDGFRβ) Imaging: Influence of Different Linkers. *Molecules* **2020**, *26*, doi:10.3390/molecules26010041.
94. Effendi, N.; Ogawa, K.; Mishiro, K.; Takarada, T.; Yamada, D.; Kitamura, Y.; Shiba, K.; Maeda, T.; Odani, A. Synthesis and evaluation of radioiodinated 1-{2-[5-(2-methoxyethoxy)-1H-benzo[d]imidazol-1-yl]quinolin-8-yl}piperidin-4-amine derivatives for platelet-derived growth factor receptor β (PDGFRβ) imaging. *Bioorg Med Chem* **2017**, *25*, 5576-5585, doi:10.1016/j.bmc.2017.08.025.
95. Gaykema, S.B.; Brouwers, A.H.; Lub-de Hooge, M.N.; Pleijhuis, R.G.; Timmer-Bosscha, H.; Pot, L.; van Dam, G.M.; van der Meulen, S.B.; de Jong, J.R.; Bart, J.; et al. 89Zr-bevacizumab PET imaging in primary breast cancer. *J Nucl Med* **2013**, *54*, 1014-1018, doi:10.2967/jnumed.112.117218.
96. Oosting, S.F.; Brouwers, A.H.; van Es, S.C.; Nagengast, W.B.; Oude Munnink, T.H.; Lub-de Hooge, M.N.; Hollema, H.; de Jong, J.R.; de Jong, I.J.; de Haas, S.; et al. 89Zr-bevacizumab PET visualizes heterogeneous tracer accumulation in tumor lesions of renal cell carcinoma patients and differential effects of antiangiogenic treatment. *J Nucl Med* **2015**, *56*, 63-69, doi:10.2967/jnumed.114.144840.
97. Jansen, M.H.; Veldhuijzen van Zanten, S.E.M.; van Vuurden, D.G.; Huisman, M.C.; Vugts, D.J.; Hoekstra, O.S.; van Dongen, G.A.; Kaspers, G.L. Molecular Drug Imaging: (89)Zr-Bevacizumab PET in Children with Diffuse Intrinsic Pontine Glioma. *J Nucl Med* **2017**, *58*, 711-716, doi:10.2967/jnumed.116.180216.
98. Jansen, M.H.; Lagerweij, T.; Sewing, A.C.; Vugts, D.J.; van Vuurden, D.G.; Molthoff, C.F.; Caretti, V.; Veringa, S.J.; Petersen, N.; Carcaboso, A.M.; et al. Bevacizumab Targeting Diffuse Intrinsic Pontine Glioma: Results of 89Zr-Bevacizumab PET Imaging in Brain Tumor Models. *Mol Cancer Ther* **2016**, *15*, 2166-2174, doi:10.1158/1535-7163.mct-15-0558.
99. Veldhuijzen van Zanten, S.E.M.; Sewing, A.C.P.; van Lingen, A.; Hoekstra, O.S.; Wesseling, P.; Meel, M.H.; van Vuurden, D.G.; Kaspers, G.J.L.; Hulleman, E.; Bugiani, M. Multiregional Tumor Drug-Uptake Imaging by PET and Microvascular Morphology in End-Stage Diffuse Intrinsic Pontine Glioma. *J Nucl Med* **2018**, *59*, 612-615, doi:10.2967/jnumed.117.197897.
100. Kameswaran, M.; Sarma, H.D.; Dash, A. Preclinical evaluation of (131)I-Bevacizumab - A prospective agent for radioimmunotherapy in VEGF expressing cancers. *Appl Radiat Isot* **2017**, *123*, 109-113, doi:10.1016/j.apradiso.2017.02.024.
101. Stollman, T.H.; Scheer, M.G.; Leenders, W.P.; Verrijp, K.C.; Soede, A.C.; Oyen, W.J.; Ruers, T.J.; Boerman, O.C. Specific imaging of VEGF-A expression with radiolabeled anti-VEGF monoclonal antibody. *Int J Cancer* **2008**, *122*, 2310-2314, doi:10.1002/ijc.23404.
102. van Es, S.C.; Brouwers, A.H.; Mahesh, S.V.K.; Leliveld-Kors, A.M.; de Jong, I.J.; Lub-de Hooge, M.N.; de Vries, E.G.E.; Gietema, J.A.; Oosting, S.F. (89)Zr-Bevacizumab PET: Potential Early Indicator of Everolimus Efficacy in Patients with Metastatic Renal Cell Carcinoma. *J Nucl Med* **2017**, *58*, 905-910, doi:10.2967/jnumed.116.183475.
103. Nagengast, W.B.; Lub-de Hooge, M.N.; Oosting, S.F.; den Dunnen, W.F.; Warnders, F.J.; Brouwers, A.H.; de Jong, J.R.; Price, P.M.; Hollema, H.; Hospers, G.A.; et al. VEGF-PET imaging is a noninvasive biomarker showing differential changes in the tumor during sunitinib treatment. In *Cancer Res* 2011, 71, 143-153.
104. Laffon, E.; Marthan, R. A three-time-point method for assessing kinetic parameters of (64)Cu-labeled Ramucirumab trapping in VEGFR-2 positive lung tumors. *Phys Med* **2017**, *43*, 1-5, doi:10.1016/j.ejmp.2017.10.001.
105. Luo, H.; England, C.G.; Graves, S.A.; Sun, H.; Liu, G.; Nickles, R.J.; Cai, W. PET Imaging of VEGFR-2 Expression in Lung Cancer with 64Cu-Labeled Ramucirumab. *J Nucl Med* **2016**, *57*, 285-290, doi:10.2967/jnumed.115.166462.

106. Slobbe, P.; Poot, A.J.; Haumann, R.; Schuit, R.C.; Windhorst, A.D.; van Dongen, G.A. Two anti-angiogenic TKI-PET tracers, [(11)C]axitinib and [(11)C]nintedanib: Radiosynthesis, in vivo metabolism and initial biodistribution studies in rodents. *Nucl Med Biol* **2016**, *43*, 612-624, doi:10.1016/j.nucmedbio.2016.07.003.
107. Samén, E.; Lu, L.; Mulder, J.; Thorell, J.O.; Damberg, P.; Tegnebratt, T.; Holmgren, L.; Rundqvist, H.; Stone-Elander, S. Visualization of angiogenesis during cancer development in the polyoma middle T breast cancer model: molecular imaging with (R)-[11C]PAQ. *EJNMMI Res* **2014**, *4*, 17, doi:10.1186/2191-219x-4-17.
108. Meyer, J.P.; Edwards, K.J.; Kozlowski, P.; Backer, M.V.; Backer, J.M.; Lewis, J.S. Selective Imaging of VEGFR-1 and VEGFR-2 Using 89Zr-Labeled Single-Chain VEGF Mutants. *J Nucl Med* **2016**, *57*, 1811-1816, doi:10.2967/jnumed.116.173237.
109. Blankenberg, F.G.; Vanderheyden, J.L.; Strauss, H.W.; Tait, J.F. Radiolabeling of HYNIC-annexin V with technetium-99m for in vivo imaging of apoptosis. *Nat Protoc* **2006**, *1*, 108-110, doi:10.1038/nprot.2006.17.
110. Hu, P.H.; Pan, L.H.; Wong, P.T.; Chen, W.H.; Yang, Y.Q.; Wang, H.; Xiang, J.J.; Xu, M. (125)I-labeled anti-bFGF monoclonal antibody inhibits growth of hepatocellular carcinoma. *World J Gastroenterol* **2016**, *22*, 5033-5041, doi:10.3748/wjg.v22.i21.5033.
111. Charmsaz, S.; Al-Ejeh, F.; Yeadon, T.M.; Miller, K.J.; Smith, F.M.; Stringer, B.W.; Moore, A.S.; Lee, F.T.; Cooper, L.T.; Stylianou, C.; et al. EphA3 as a target for antibody immunotherapy in acute lymphoblastic leukemia. *Leukemia* **2017**, *31*, 1779-1787, doi:10.1038/leu.2016.371.
112. Vearing, C.; Lee, F.T.; Wimmer-Kleikamp, S.; Spirkoska, V.; To, C.; Stylianou, C.; Spanevello, M.; Brechbiel, M.; Boyd, A.W.; Scott, A.M.; et al. Concurrent binding of anti-EphA3 antibody and ephrin-A5 amplifies EphA3 signaling and downstream responses: potential as EphA3-specific tumor-targeting reagents. *Cancer Res* **2005**, *65*, 6745-6754, doi:10.1158/0008-5472.can-05-0758.
113. Liu, S.; Li, D.; Park, R.; Liu, R.; Xia, Z.; Guo, J.; Krasnoperov, V.; Gill, P.S.; Li, Z.; Shan, H.; et al. PET imaging of colorectal and breast cancer by targeting EphB4 receptor with 64Cu-labeled hAb47 and hAb131 antibodies. *J Nucl Med* **2013**, *54*, 1094-1100, doi:10.2967/jnumed.112.116822.
114. Xiong, C.; Huang, M.; Zhang, R.; Song, S.; Lu, W.; Flores, L., 2nd; Gelovani, J.; Li, C. In vivo small-animal PET/CT of EphB4 receptors using 64Cu-labeled peptide. *J Nucl Med* **2011**, *52*, 241-248, doi:10.2967/jnumed.110.081943.
115. Zhang, R.; Xiong, C.; Huang, M.; Zhou, M.; Huang, Q.; Wen, X.; Liang, D.; Li, C. Peptide-conjugated polymeric micellar nanoparticles for Dual SPECT and optical imaging of EphB4 receptors in prostate cancer xenografts. *Biomaterials* **2011**, *32*, 5872-5879, doi:10.1016/j.biomaterials.2011.04.070.
116. Ebert, K.; Wiemer, J.; Caballero, J.; Köckerling, M.; Steinbach, J.; Pietzsch, J.; Mamat, C. Development of indazolylpyrimidine derivatives as high-affine EphB4 receptor ligands and potential PET radiotracers. *Bioorg Med Chem* **2015**, *23*, 6025-6035, doi:10.1016/j.bmc.2015.06.040.
117. Mamat, C.; Mosch, B.; Neuber, C.; Köckerling, M.; Bergmann, R.; Pietzsch, J. Fluorine-18 radiolabeling and radiopharmacological characterization of a benzodioxolylpyrimidine-based radiotracer targeting the receptor tyrosine kinase EphB4. *ChemMedChem* **2012**, *7*, 1991-2003, doi:10.1002/cmdc.201200264.
118. Charmsaz, S.; Beckett, K.; Smith, F.M.; Bruedigam, C.; Moore, A.S.; Al-Ejeh, F.; Lane, S.W.; Boyd, A.W. EphA2 Is a Therapy Target in EphA2-Positive Leukemias but Is Not Essential for Normal Hematopoiesis or Leukemia. *PLoS One* **2015**, *10*, e0130692, doi:10.1371/journal.pone.0130692.
119. Cai, W.; Ebrahimnejad, A.; Chen, K.; Cao, Q.; Li, Z.B.; Tice, D.A.; Chen, X. Quantitative radioimmunoPET imaging of EphA2 in tumor-bearing mice. *Eur J Nucl Med Mol Imaging* **2007**, *34*, 2024-2036, doi:10.1007/s00259-007-0503-5.

120. Burvenich, I.J.; Parakh, S.; Gan, H.K.; Lee, F.T.; Guo, N.; Rigopoulos, A.; Lee, S.T.; Gong, S.; O'Keefe, G.J.; Tochon-Danguy, H.; et al. Molecular Imaging and Quantitation of EphA2 Expression in Xenograft Models with 89Zr-DS-8895a. *J Nucl Med* **2016**, *57*, 974-980, doi:10.2967/jnumed.115.169839.
121. ClinicalTrials.gov. Available online: https://clinicaltrials.gov/ct2/home (accessed March 2020 - May 2021).
122. Pretze, M.; Mosch, B.; Bergmann, R.; Steinbach, J.; Pietzsch, J.; Mamat, C. Radiofluorination and first radiopharmacological characterization of a SWLAY peptide-based ligand targeting EphA2. *J Labelled Comp Radiopharm* **2014**, *57*, 660-665, doi:10.1002/jlcr.3237.
123. Liu, Y.; Lan, X.; Wu, T.; Lang, J.; Jin, X.; Sun, X.; Wen, Q.; An, R. (99m)Tc-labeled SWL specific peptide for targeting EphA2 receptor. *Nucl Med Biol* **2014**, *41*, 450-456, doi:10.1016/j.nucmedbio.2014.03.020.
124. Pretze, M.; Neuber, C.; Kinski, E.; Belter, B.; Köckerling, M.; Caflisch, A.; Steinbach, J.; Pietzsch, J.; Mamat, C. Synthesis, radiolabelling and initial biological characterisation of (18)F-labelled xanthine derivatives for PET imaging of Eph receptors. *Org Biomol Chem* **2020**, *18*, 3104-3116, doi:10.1039/d0ob00391c.
125. Pretze, M.; Kuchar, M.; Bergmann, R.; Steinbach, J.; Pietzsch, J.; Mamat, C. An efficient bioorthogonal strategy using CuAAC click chemistry for radiofluorinations of SNEW peptides and the role of copper depletion. *ChemMedChem* **2013**, *8*, 935-945, doi:10.1002/cmdc.201300053.
126. Heskamp, S.; van Laarhoven, H.W.; Molkenboer-Kuenen, J.D.; Franssen, G.M.; Versleijen-Jonkers, Y.M.; Oyen, W.J.; van der Graaf, W.T.; Boerman, O.C. ImmunoSPECT and immunoPET of IGF-1R expression with the radiolabeled antibody R1507 in a triple-negative breast cancer model. *J Nucl Med* **2010**, *51*, 1565-1572, doi:10.2967/jnumed.110.075648.
127. Heskamp, S.; van Laarhoven, H.W.; Molkenboer-Kuenen, J.D.; Bouwman, W.H.; van der Graaf, W.T.; Oyen, W.J.; Boerman, O.C. Optimization of IGF-1R SPECT/CT imaging using 111In-labeled F(ab')2 and Fab fragments of the monoclonal antibody R1507. *Mol Pharm* **2012**, *9*, 2314-2321, doi:10.1021/mp300232n.
128. Fleuren, E.D.; Versleijen-Jonkers, Y.M.; van de Luijtgaarden, A.C.; Molkenboer-Kuenen, J.D.; Heskamp, S.; Roeffen, M.H.; van Laarhoven, H.W.; Houghton, P.J.; Oyen, W.J.; Boerman, O.C.; et al. Predicting IGF-1R therapy response in bone sarcomas: immuno-SPECT imaging with radiolabeled R1507. *Clin Cancer Res* **2011**, *17*, 7693-7703, doi:10.1158/1078-0432.ccr-11-1488.
129. Sun, B.F.; Kobayashi, H.; Le, N.; Yoo, T.M.; Drumm, D.; Paik, C.H.; McAfee, J.G.; Carrasquillo, J.A. Effects of insulinlike growth factor binding proteins on insulinlike growth factor-I biodistribution in tumor-bearing nude mice. *J Nucl Med* **2000**, *41*, 318-326.
130. Sun, B.F.; Kobayashi, H.; Le, N.; Yoo, T.M.; Drumm, D.; Paik, C.H.; McAfee, J.G.; Carrasquillo, J.A. Biodistribution of 125I-labeled des(1-3) insulin-like growth factor I in tumor-bearing nude mice and its in vitro catabolism. *Cancer Res* **1997**, *57*, 2754-2759.
131. England, C.G.; Kamkaew, A.; Im, H.J.; Valdovinos, H.F.; Sun, H.; Hernandez, R.; Cho, S.Y.; Dunphy, E.J.; Lee, D.S.; Barnhart, T.E.; et al. ImmunoPET Imaging of Insulin-Like Growth Factor 1 Receptor in a Subcutaneous Mouse Model of Pancreatic Cancer. *Mol Pharm* **2016**, *13*, 1958-1966, doi:10.1021/acs.molpharmaceut.6b00132.
132. Cornelissen, B.; McLarty, K.; Kersemans, V.; Reilly, R.M. The level of insulin growth factor-1 receptor expression is directly correlated with the tumor uptake of (111)In-IGF-1(E3R) in vivo and the clonogenic survival of breast cancer cells exposed in vitro to trastuzumab (Herceptin). *Nucl Med Biol* **2008**, *35*, 645-653, doi:10.1016/j.nucmedbio.2008.05.010.

133. Tolmachev, V.; Malmberg, J.; Hofström, C.; Abrahmsén, L.; Bergman, T.; Sjöberg, A.; Sandström, M.; Gräslund, T.; Orlova, A. Imaging of insulinlike growth factor type 1 receptor in prostate cancer xenografts using the affibody molecule 111In-DOTA-ZIGF1R:4551. *J Nucl Med* **2012**, *53*, 90-97, doi:10.2967/jnumed.111.090829.
134. Orlova, A.; Hofström, C.; Strand, J.; Varasteh, Z.; Sandstrom, M.; Andersson, K.; Tolmachev, V.; Gräslund, T. [99mTc(CO)3]+-(HE)3-ZIGF1R:4551, a new Affibody conjugate for visualization of insulin-like growth factor-1 receptor expression in malignant tumours. *Eur J Nucl Med Mol Imaging* **2013**, *40*, 439-449, doi:10.1007/s00259-012-2284-8.
135. Mitran, B.; Altai, M.; Hofström, C.; Honarvar, H.; Sandström, M.; Orlova, A.; Tolmachev, V.; Gräslund, T. Evaluation of 99mTc-Z IGF1R:4551-GGGC affibody molecule, a new probe for imaging of insulin-like growth factor type 1 receptor expression. *Amino Acids* **2015**, *47*, 303-315, doi:10.1007/s00726-014-1859-z.
136. Majo, V.J.; Arango, V.; Simpson, N.R.; Prabhakaran, J.; Kassir, S.A.; Underwood, M.D.; Bakalian, M.; Canoll, P.; John Mann, J.; Dileep Kumar, J.S. Synthesis and in vitro evaluation of [18F]BMS-754807: a potential PET ligand for IGF-1R. *Bioorg Med Chem Lett* **2013**, *23*, 4191-4194, doi:10.1016/j.bmcl.2013.05.026.
137. Prabhakaran, J.; Dewey, S.L.; McClure, R.; Simpson, N.R.; Tantawy, M.N.; Mann, J.J.; Pham, W.; Kumar, J.S.D. In vivo evaluation of IGF1R/IR PET ligand [(18)F]BMS-754807 in rodents. *Bioorg Med Chem Lett* **2017**, *27*, 941-943, doi:10.1016/j.bmcl.2016.12.086.
138. Makino, A.; Arai, T.; Hirata, M.; Ono, M.; Ohmomo, Y.; Saji, H. Development of novel PET probes targeting phosphatidylinositol 3-kinase (PI3K) in tumors. *Nucl Med Biol* **2016**, *43*, 101-107, doi:10.1016/j.nucmedbio.2015.09.008.
139. Peng, Z.; Maxwell, D.S.; Sun, D.; Bhanu Prasad, B.A.; Pal, A.; Wang, S.; Balatoni, J.; Ghosh, P.; Lim, S.T.; Volgin, A.; et al. Imatinib analogs as potential agents for PET imaging of Bcr-Abl and c-KIT expression at a kinase level. *Bioorg Med Chem* **2014**, *22*, 623-632, doi:10.1016/j.bmc.2013.10.040.
140. Kil, K.E.; Ding, Y.S.; Lin, K.S.; Alexoff, D.; Kim, S.W.; Shea, C.; Xu, Y.; Muench, L.; Fowler, J.S. Synthesis and positron emission tomography studies of carbon-11-labeled imatinib (Gleevec). *Nucl Med Biol* **2007**, *34*, 153-163, doi:10.1016/j.nucmedbio.2006.11.004.
141. Asakawa, C.; Ogawa, M.; Kumata, K.; Fujinaga, M.; Kato, K.; Yamasaki, T.; Yui, J.; Kawamura, K.; Hatori, A.; Fukumura, T.; et al. [11C]sorafenib: radiosynthesis and preliminary PET study of brain uptake in P-gp/Bcrp knockout mice. *Bioorg Med Chem Lett* **2011**, *21*, 2220-2223, doi:10.1016/j.bmcl.2011.03.002.
142. Lagas, J.S.; van Waterschoot, R.A.; Sparidans, R.W.; Wagenaar, E.; Beijnen, J.H.; Schinkel, A.H. Breast cancer resistance protein and P-glycoprotein limit sorafenib brain accumulation. *Mol Cancer Ther* **2010**, *9*, 319-326, doi:10.1158/1535-7163.mct-09-0663.
143. Poot, A.J.; van der Wildt, B.; Stigter-van Walsum, M.; Rongen, M.; Schuit, R.C.; Hendrikse, N.H.; Eriksson, J.; van Dongen, G.A.; Windhorst, A.D. [^{11}C]Sorafenib: radiosynthesis and preclinical evaluation in tumor-bearing mice of a new TKI-PET tracer. *Nucl Med Biol* **2013**, *40*, 488-497, doi:10.1016/j.nucmedbio.2013.02.002.
144. Saleem, A.; Searle, G.E.; Kenny, L.M.; Huiban, M.; Kozlowski, K.; Waldman, A.D.; Woodley, L.; Palmieri, C.; Lowdell, C.; Kaneko, T.; et al. Lapatinib access into normal brain and brain metastases in patients with Her-2 overexpressing breast cancer. *EJNMMI Res* **2015**, *5*, 30, doi:10.1186/s13550-015-0103-5.

145. Basuli, F.; Wu, H.; Li, C.; Shi, Z.-D.; Sulima, A.; Griffiths, G.L. A first synthesis of 18F-radiolabeled lapatinib: a potential tracer for positron emission tomographic imaging of ErbB1/ErbB2 tyrosine kinase activity. *Journal of Labelled Compounds and Radiopharmaceuticals* **2011**, *54*, 633-636, doi:https://doi.org/10.1002/jlcr.1898.
146. Caballero, J.; Muñoz, C.; Alzate-Morales, J.H.; Cunha, S.; Gano, L.; Bergmann, R.; Steinbach, J.; Kniess, T. Synthesis, in silico, in vitro, and in vivo investigation of 5-[^{11}C]methoxy-substituted sunitinib, a tyrosine kinase inhibitor of VEGFR-2. *Eur J Med Chem* **2012**, *58*, 272-280, doi:10.1016/j.ejmech.2012.10.020.
147. Lien, V.T.; Celen, S.; Nuruddin, S.; Attili, B.; Doumont, G.; Van Simaeys, G.; Bormans, G.; Klaveness, J.; Olberg, D.E. Preclinical evaluation of [(18)F]cabozantinib as a PET imaging agent in a prostate cancer mouse model. *Nucl Med Biol* **2020**, *93*, 74-80, doi:10.1016/j.nucmedbio.2020.12.002.
148. Gao, M.; Lola, C.M.; Wang, M.; Miller, K.D.; Sledge, G.W.; Zheng, Q.H. Radiosynthesis of [11C]Vandetanib and [11C]chloro-Vandetanib as new potential PET agents for imaging of VEGFR in cancer. *Bioorg Med Chem Lett* **2011**, *21*, 3222-3226, doi:10.1016/j.bmcl.2011.04.049.
149. Cavaliere, A.; Sun, S.; Lee, S.; Bodner, J.; Li, Z.; Huang, Y.; Moores, S.L.; Marquez-Nostra, B. Development of [(89)Zr]ZrDFO-amivantamab bispecific to EGFR and c-MET for PET imaging of triple-negative breast cancer. *Eur J Nucl Med Mol Imaging* **2021**, *48*, 383-394, doi:10.1007/s00259-020-04978-6.
150. Ye, Y.E.; Woodward, C.N.; Narasimhan, N.I. Absorption, metabolism, and excretion of [(14)C]ponatinib after a single oral dose in humans. *Cancer Chemother Pharmacol* **2017**, *79*, 507-518, doi:10.1007/s00280-017-3240-x.
